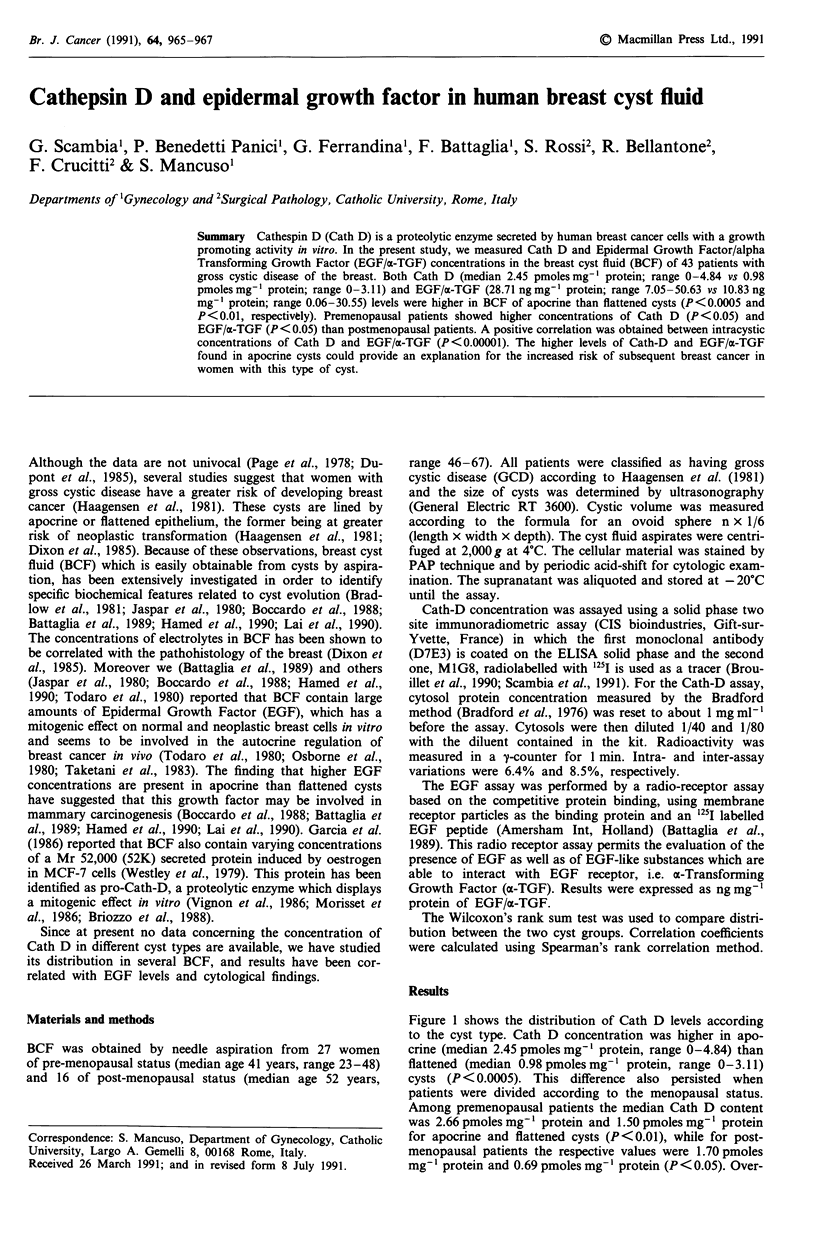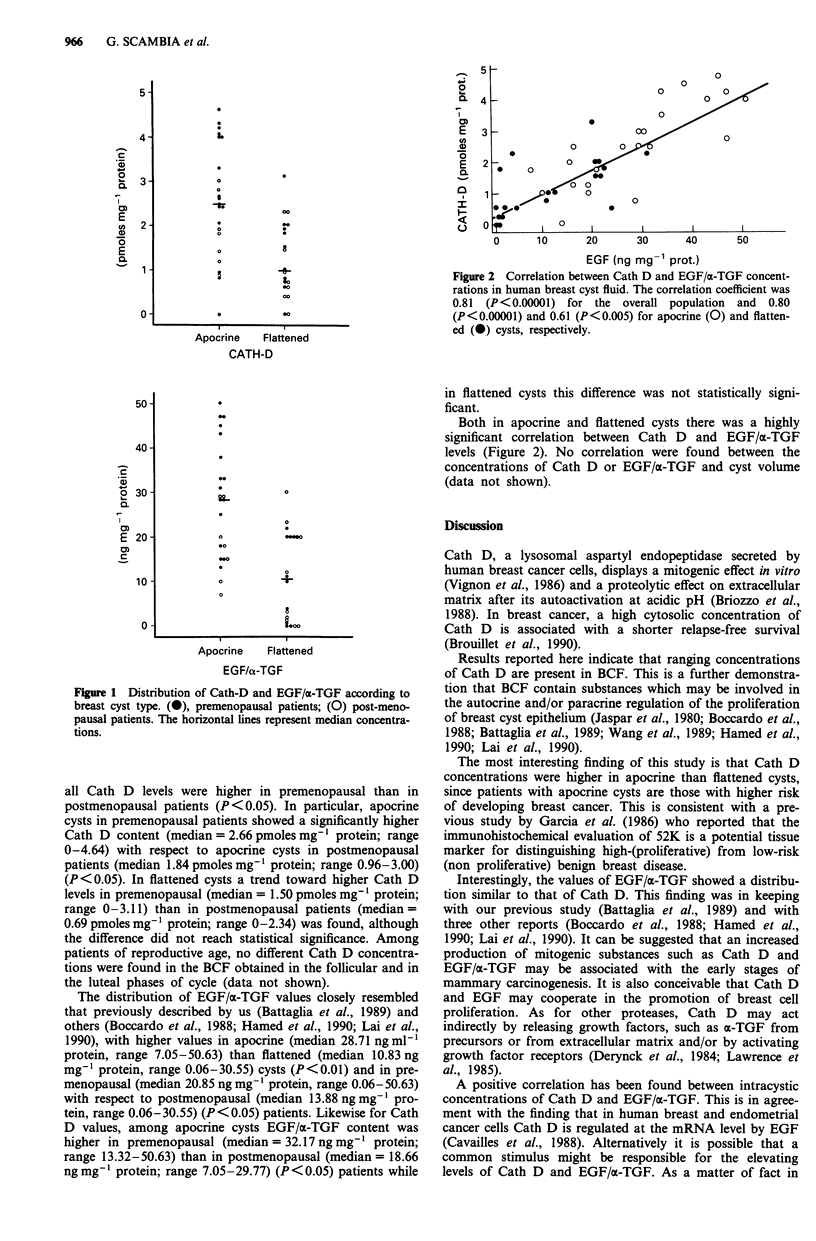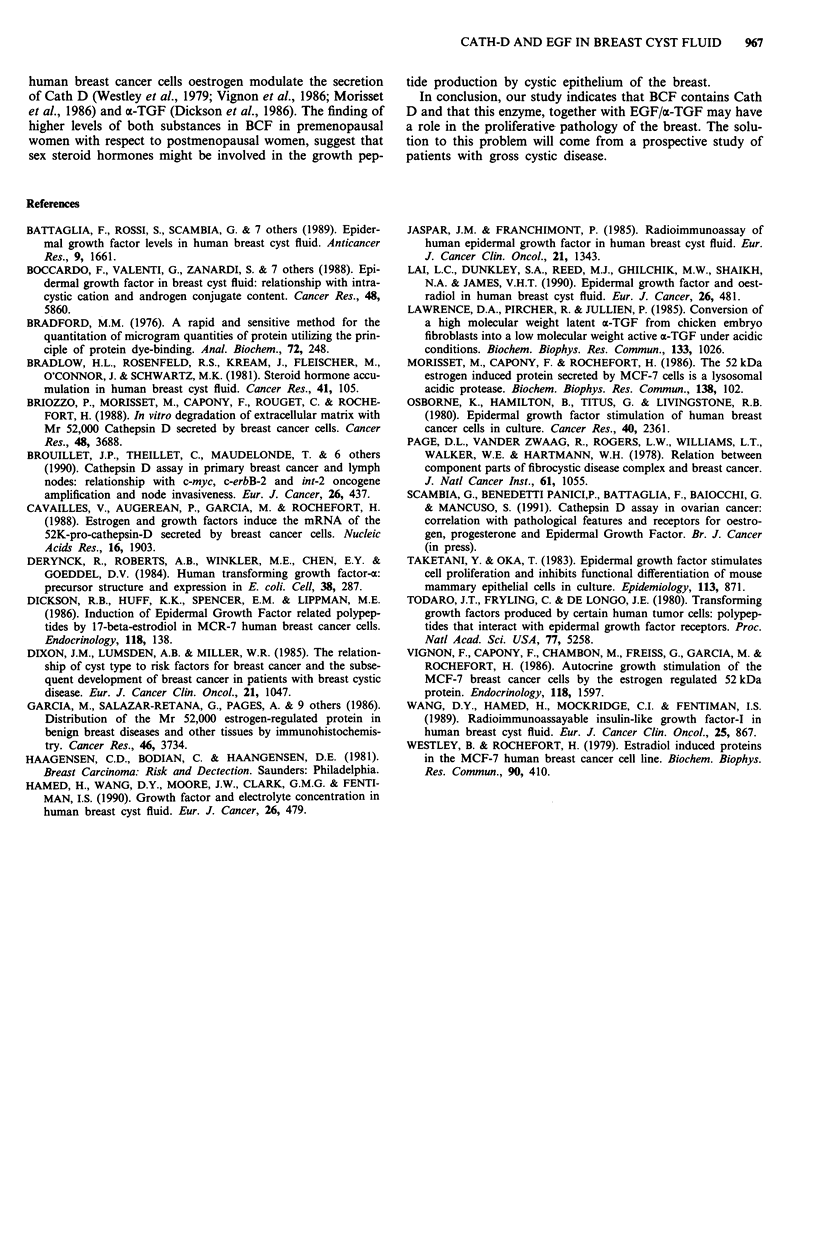# Cathepsin D and epidermal growth factor in human breast cyst fluid.

**DOI:** 10.1038/bjc.1991.437

**Published:** 1991-11

**Authors:** G. Scambia, P. Benedetti Panici, G. Ferrandina, F. Battaglia, S. Rossi, R. Bellantone, F. Crucitti, S. Mancuso

**Affiliations:** Department of Gynecology, Catholic University, Rome, Italy.

## Abstract

Cathespin D (Cath D) is a proteolytic enzyme secreted by human breast cancer cells with a growth promoting activity in vitro. In the present study, we measured Cath D and Epidermal Growth Factor/alpha Transforming Growth Factor (EGF/alpha-TGF) concentrations in the breast cyst fluid (BCF) of 43 patients with gross cystic disease of the breast. Both Cath D (median 2.45 pmoles mg-1 protein; range 0-4.84 vs 0.98 pmoles mg-1 protein; range 0-3.11) and EGF/alpha-TGF (28.71 ng mg-1 protein; range 7.05-50.63 vs 10.83 ng mg-1 protein; range 0.06-30.55) levels were higher in BCF of apocrine than flattened cysts (P less than 0.0005 and P less than 0.01, respectively). Premenopausal patients showed higher concentrations of Cath D (P less than 0.05) and EGF/alpha-TGF (P less than 0.05) than postmenopausal patients. A positive correlation was obtained between intracystic concentrations of Cath D and EGF/alpha-TGF (P less than 0.00001). The higher levels of Cath-D and EGF/alpha-TGF found in apocrine cysts could provide an explanation for the increased risk of subsequent breast cancer in women with this type of cyst.


*Br. J. Cancer* (1991), **64**, 965–967 © Macmillan Press Ltd., 1991

### Cathepsin D and epidermal growth factor in human breast cyst fluid

G. Scambia[1], P. Benedetti Panici[1], G. Ferrandina[1], F. Battaglia[1], S. Rossi[2], R. Bellantone[2], F. Crucitti[2] & S. Mancuso[1]

*Departments of [1]Gynecology and [2]Surgical Pathology, Catholic University, Rome, Italy*

**Summary** Cathespin D (Cath D) is a proteolytic enzyme secreted by human breast cancer cells with a growth promoting activity *in vitro*. In the present study, we measured Cath D and Epidermal Growth Factor/alpha Transforming Growth Factor (EGF/α-TGF) concentrations in the breast cyst fluid (BCF) of 43 patients with gross cystic disease of the breast. Both Cath D (median 2.45 pmoles $mg^{-1}$ protein; range 0–4.84 *vs* 0.98 pmoles $mg^{-1}$ protein; range 0–3.11) and EGF/α-TGF (28.71 ng $mg^{-1}$ protein; range 7.05–50.63 *vs* 10.83 ng $mg^{-1}$ protein; range 0.06–30.55) levels were higher in BCF of apocrine than flattened cysts ($P<0.0005$ and $P<0.01$, respectively). Premenopausal patients showed higher concentrations of Cath D ($P<0.05$) and EGF/α-TGF ($P<0.05$) than postmenopausal patients. A positive correlation was obtained between intracystic concentrations of Cath D and EGF/α-TGF ($P<0.00001$). The higher levels of Cath-D and EGF/α-TGF found in apocrine cysts could provide an explanation for the increased risk of subsequent breast cancer in women with this type of cyst.

Although the data are not univocal (Page *et al*., 1978; Dupont *et al*., 1985), several studies suggest that women with gross cystic disease have a greater risk of developing breast cancer (Haagensen *et al*., 1981). These cysts are lined by apocrine or flattened epithelium, the former being at greater risk of neoplastic transformation (Haagensen *et al*., 1981; Dixon *et al*., 1985). Because of these observations, breast cyst fluid (BCF) which is easily obtainable from cysts by aspiration, has been extensively investigated in order to identify specific biochemical features related to cyst evolution (Bradlow *et al*., 1981; Jaspar *et al*., 1980; Boccardo *et al*., 1988; Battaglia *et al*., 1989; Hamed *et al*., 1990; Lai *et al*., 1990). The concentrations of electrolytes in BCF has been shown to be correlated with the pathohistology of the breast (Dixon *et al*., 1985). Moreover we (Battaglia *et al*., 1989) and others (Jaspar *et al*., 1980; Boccardo *et al*., 1988; Hamed *et al*., 1990; Todaro *et al*., 1980) reported that BCF contain large amounts of Epidermal Growth Factor (EGF), which has a mitogenic effect on normal and neoplastic breast cells *in vitro* and seems to be involved in the autocrine regulation of breast cancer *in vivo* (Todaro *et al*., 1980; Osborne *et al*., 1980; Taketani *et al*., 1983). The finding that higher EGF concentrations are present in apocrine than flattened cysts have suggested that this growth factor may be involved in mammary carcinogenesis (Boccardo *et al*., 1988; Battaglia *et al*., 1989; Hamed *et al*., 1990; Lai *et al*., 1990). Garcia *et al*. (1986) reported that BCF also contain varying concentrations of a Mr 52,000 (52K) secreted protein induced by oestrogen in MCF-7 cells (Westley *et al*., 1979). This protein has been identified as pro-Cath-D, a proteolytic enzyme which displays a mitogenic effect *in vitro* (Vignon *et al*., 1986; Morisset *et al*., 1986; Briozzo *et al*., 1988).

Since at present no data concerning the concentration of Cath D in different cyst types are available, we have studied its distribution in several BCF, and results have been correlated with EGF levels and cytological findings.

### Materials and methods

BCF was obtained by needle aspiration from 27 women of pre-menopausal status (median age 41 years, range 23–48) and 16 of post-menopausal status (median age 52 years, range 46–67). All patients were classified as having gross cystic disease (GCD) according to Haagensen *et al*. (1981) and the size of cysts was determined by ultrasonography (General Electric RT 3600). Cystic volume was measured according to the formula for an ovoid sphere $n \times 1/6$ (length × width × depth). The cyst fluid aspirates were centrifuged at 2,000 *g* at 4°C. The cellular material was stained by PAP technique and by periodic acid-shift for cytologic examination. The supranatant was aliquoted and stored at −20°C until the assay.

Cath-D concentration was assayed using a solid phase two site immunoradiometric assay (CIS bioindustries, Gift-sur-Yvette, France) in which the first monoclonal antibody (D7E3) is coated on the ELISA solid phase and the second one, M1G8, radiolabelled with $^{125}I$ is used as a tracer (Brouillet *et al*., 1990; Scambia *et al*., 1991). For the Cath-D assay, cytosol protein concentration measured by the Bradford method (Bradford *et al*., 1976) was reset to about 1 mg $ml^{-1}$ before the assay. Cytosols were then diluted 1/40 and 1/80 with the diluent contained in the kit. Radioactivity was measured in a γ-counter for 1 min. Intra- and inter-assay variations were 6.4% and 8.5%, respectively.

The EGF assay was performed by a radio-receptor assay based on the competitive protein binding, using membrane receptor particles as the binding protein and an $^{125}I$ labelled EGF peptide (Amersham Int, Holland) (Battaglia *et al*., 1989). This radio receptor assay permits the evaluation of the presence of EGF as well as of EGF-like substances which are able to interact with EGF receptor, i.e. α-Transforming Growth Factor (α-TGF). Results were expressed as ng $mg^{-1}$ protein of EGF/α-TGF.

The Wilcoxon's rank sum test was used to compare distribution between the two cyst groups. Correlation coefficients were calculated using Spearman's rank correlation method.

### Results

Figure 1 shows the distribution of Cath D levels according to the cyst type. Cath D concentration was higher in apocrine (median 2.45 pmoles $mg^{-1}$ protein, range 0–4.84) than flattened (median 0.98 pmoles $mg^{-1}$ protein, range 0–3.11) cysts ($P<0.0005$). This difference also persisted when patients were divided according to the menopausal status. Among premenopausal patients the median Cath D content was 2.66 pmoles $mg^{-1}$ protein and 1.50 pmoles $mg^{-1}$ protein for apocrine and flattened cysts ($P<0.01$), while for post-menopausal patients the respective values were 1.70 pmoles $mg^{-1}$ protein and 0.69 pmoles $mg^{-1}$ protein ($P<0.05$). Over-

Correspondence: S. Mancuso, Department of Gynecology, Catholic University, Largo A. Gemelli 8, 00168 Rome, Italy.
Received 26 March 1991; and in revised form 8 July 1991.

**Figure 1** Distribution of Cath-D and EGF/α-TGF according to breast cyst type. (●), premenopausal patients; (O) post-menopausal patients. The horizontal lines represent median concentrations.

**Figure 2** Correlation between Cath D and EGF/α-TGF concentrations in human breast cyst fluid. The correlation coefficient was 0.81 ($P<0.00001$) for the overall population and 0.80 ($P<0.00001$) and 0.61 ($P<0.005$) for apocrine (O) and flattened (●) cysts, respectively.

all Cath D levels were higher in premenopausal than in postmenopausal patients ($P<0.05$). In particular, apocrine cysts in premenopausal patients showed a significantly higher Cath D content (median = 2.66 pmoles mg$^{-1}$ protein; range 0–4.64) with respect to apocrine cysts in postmenopausal patients (median 1.84 pmoles mg$^{-1}$ protein; range 0.96–3.00) ($P<0.05$). In flattened cysts a trend toward higher Cath D levels in premenopausal (median = 1.50 pmoles mg$^{-1}$ protein; range 0–3.11) than in postmenopausal patients (median = 0.69 pmoles mg$^{-1}$ protein; range 0–2.34) was found, although the difference did not reach statistical significance. Among patients of reproductive age, no different Cath D concentrations were found in the BCF obtained in the follicular and in the luteal phases of cycle (data not shown).

The distribution of EGF/α-TGF values closely resembled that previously described by us (Battaglia *et al.*, 1989) and others (Boccardo *et al.*, 1988; Hamed *et al.*, 1990; Lai *et al.*, 1990), with higher values in apocrine (median 28.71 ng ml$^{-1}$ protein, range 7.05–50.63) than flattened (median 10.83 ng mg$^{-1}$ protein, range 0.06–30.55) cysts ($P<0.01$) and in premenopausal (median 20.85 ng mg$^{-1}$ protein, range 0.06–50.63) with respect to postmenopausal (median 13.88 ng mg$^{-1}$ protein, range 0.06–30.55) ($P<0.05$) patients. Likewise for Cath D values, among apocrine cysts EGF/α-TGF content was higher in premenopausal (median = 32.17 ng mg$^{-1}$ protein; range 13.32–50.63) than in postmenopausal (median = 18.66 ng mg$^{-1}$ protein; range 7.05–29.77) ($P<0.05$) patients while in flattened cysts this difference was not statistically significant.

Both in apocrine and flattened cysts there was a highly significant correlation between Cath D and EGF/α-TGF levels (Figure 2). No correlation were found between the concentrations of Cath D or EGF/α-TGF and cyst volume (data not shown).

### Discussion

Cath D, a lysosomal aspartyl endopeptidase secreted by human breast cancer cells, displays a mitogenic effect *in vitro* (Vignon *et al.*, 1986) and a proteolytic effect on extracellular matrix after its autoactivation at acidic pH (Briozzo *et al.*, 1988). In breast cancer, a high cytosolic concentration of Cath D is associated with a shorter relapse-free survival (Brouillet *et al.*, 1990).

Results reported here indicate that ranging concentrations of Cath D are present in BCF. This is a further demonstration that BCF contain substances which may be involved in the autocrine and/or paracrine regulation of the proliferation of breast cyst epithelium (Jaspar *et al.*, 1980; Boccardo *et al.*, 1988; Battaglia *et al.*, 1989; Wang *et al.*, 1989; Hamed *et al.*, 1990; Lai *et al.*, 1990).

The most interesting finding of this study is that Cath D concentrations were higher in apocrine than flattened cysts, since patients with apocrine cysts are those with higher risk of developing breast cancer. This is consistent with a previous study by Garcia *et al.* (1986) who reported that the immunohistochemical evaluation of 52K is a potential tissue marker for distinguishing high-(proliferative) from low-risk (non proliferative) benign breast disease.

Interestingly, the values of EGF/α-TGF showed a distribution similar to that of Cath D. This finding was in keeping with our previous study (Battaglia *et al.*, 1989) and with three other reports (Boccardo *et al.*, 1988; Hamed *et al.*, 1990; Lai *et al.*, 1990). It can be suggested that an increased production of mitogenic substances such as Cath D and EGF/α-TGF may be associated with the early stages of mammary carcinogenesis. It is also conceivable that Cath D and EGF may cooperate in the promotion of breast cell proliferation. As for other proteases, Cath D may act indirectly by releasing growth factors, such as α-TGF from precursors or from extracellular matrix and/or by activating growth factor receptors (Derynck *et al.*, 1984; Lawrence *et al.*, 1985).

A positive correlation has been found between intracystic concentrations of Cath D and EGF/α-TGF. This is in agreement with the finding that in human breast and endometrial cancer cells Cath D is regulated at the mRNA level by EGF (Cavailles *et al.*, 1988). Alternatively it is possible that a common stimulus might be responsible for the elevating levels of Cath D and EGF/α-TGF. As a matter of fact in human breast cancer cells oestrogen modulate the secretion of Cath D (Westley *et al.*, 1979; Vignon *et al.*, 1986; Morisset *et al.*, 1986) and α-TGF (Dickson *et al.*, 1986). The finding of higher levels of both substances in BCF in premenopausal women with respect to postmenopausal women, suggest that sex steroid hormones might be involved in the growth peptide production by cystic epithelium of the breast.

In conclusion, our study indicates that BCF contains Cath D and that this enzyme, together with EGF/α-TGF may have a role in the proliferative pathology of the breast. The solution to this problem will come from a prospective study of patients with gross cystic disease.

### References

BATTAGLIA, F., ROSSI, S., SCAMBIA, G. & 7 others (1989). Epidermal growth factor levels in human breast cyst fluid. *Anticancer Res.*, **9**, 1661.

BOCCARDO, F., VALENTI, G., ZANARDI, S. & 7 others (1988). Epidermal growth factor in breast cyst fluid: relationship with intracystic cation and androgen conjugate content. *Cancer Res.*, **48**, 5860.

BRADFORD, M.M. (1976). A rapid and sensitive method for the quantitation of microgram quantities of protein utilizing the principle of protein dye-binding. *Anal. Biochem.*, **72**, 248.

BRADLOW, H.L., ROSENFELD, R.S., KREAM, J., FLEISCHER, M., O'CONNOR, J. & SCHWARTZ, M.K. (1981). Steroid hormone accumulation in human breast cyst fluid. *Cancer Res.*, **41**, 105.

BRIOZZO, P., MORISSET, M., CAPONY, F., ROUGET, C. & ROCHEFORT, H. (1988). *In vitro* degradation of extracellular matrix with Mr 52,000 Cathepsin D secreted by breast cancer cells. *Cancer Res.*, **48**, 3688.

BROUILLET, J.P., THEILLET, C., MAUDELONDE, T. & 6 others (1990). Cathepsin D assay in primary breast cancer and lymph nodes: relationship with c-*myc*, c-*erb*B-2 and *int*-2 oncogene amplification and node invasiveness. *Eur. J. Cancer*, **26**, 437.

CAVAILLES, V., AUGEREAN, P., GARCIA, M. & ROCHEFORT, H. (1988). Estrogen and growth factors induce the mRNA of the 52K-pro-cathepsin-D secreted by breast cancer cells. *Nucleic Acids Res.*, **16**, 1903.

DERYNCK, R., ROBERTS, A.B., WINKLER, M.E., CHEN, E.Y. & GOEDDEL, D.V. (1984). Human transforming growth factor-α: precursor structure and expression in *E. coli*. *Cell*, **38**, 287.

DICKSON, R.B., HUFF, K.K., SPENCER, E.M. & LIPPMAN, M.E. (1986). Induction of Epidermal Growth Factor related polypeptides by 17-beta-estrodiol in MCR-7 human breast cancer cells. *Endocrinology*, **118**, 138.

DIXON, J.M., LUMSDEN, A.B. & MILLER, W.R. (1985). The relationship of cyst type to risk factors for breast cancer and the subsequent development of breast cancer in patients with breast cystic disease. *Eur. J. Cancer Clin. Oncol.*, **21**, 1047.

GARCIA, M., SALAZAR-RETANA, G., PAGES, A. & 9 others (1986). Distribution of the Mr 52,000 estrogen-regulated protein in benign breast diseases and other tissues by immunohistochemistry. *Cancer Res.*, **46**, 3734.

HAAGENSEN, C.D., BODIAN, C. & HAANGENSEN, D.E. (1981). *Breast Carcinoma: Risk and Dectection*. Saunders: Philadelphia.

HAMED, H., WANG, D.Y., MOORE, J.W., CLARK, G.M.G. & FENTIMAN, I.S. (1990). Growth factor and electrolyte concentration in human breast cyst fluid. *Eur. J. Cancer*, **26**, 479.

JASPAR, J.M. & FRANCHIMONT, P. (1985). Radioimmunoassay of human epidermal growth factor in human breast cyst fluid. *Eur. J. Cancer Clin. Oncol.*, **21**, 1343.

LAI, L.C., DUNKLEY, S.A., REED, M.J., GHILCHIK, M.W., SHAIKH, N.A. & JAMES, V.H.T. (1990). Epidermal growth factor and oestradiol in human breast cyst fluid. *Eur. J. Cancer*, **26**, 481.

LAWRENCE, D.A., PIRCHER, R. & JULLIEN, P. (1985). Conversion of a high molecular weight latent α-TGF from chicken embryo fibroblasts into a low molecular weight active α-TGF under acidic conditions. *Biochem. Biophys. Res. Commun.*, **133**, 1026.

MORISSET, M., CAPONY, F. & ROCHEFORT, H. (1986). The 52 kDa estrogen induced protein secreted by MCF-7 cells is a lysosomal acidic protease. *Biochem. Biophys. Res. Commun.*, **138**, 102.

OSBORNE, K., HAMILTON, B., TITUS, G. & LIVINGSTONE, R.B. (1980). Epidermal growth factor stimulation of human breast cancer cells in culture. *Cancer Res.*, **40**, 2361.

PAGE, D.L., VANDER ZWAAG, R., ROGERS, L.W., WILLIAMS, L.T., WALKER, W.E. & HARTMANN, W.H. (1978). Relation between component parts of fibrocystic disease complex and breast cancer. *J. Natl Cancer Inst.*, **61**, 1055.

SCAMBIA, G., BENEDETTI PANICI,P., BATTAGLIA, F., BAIOCCHI, G. & MANCUSO, S. (1991). Cathepsin D assay in ovarian cancer: correlation with pathological features and receptors for oestrogen, progesterone and Epidermal Growth Factor. *Br. J. Cancer* (in press).

TAKETANI, Y. & OKA, T. (1983). Epidermal growth factor stimulates cell proliferation and inhibits functional differentiation of mouse mammary epithelial cells in culture. *Epidemiology*, **113**, 871.

TODARO, J.T., FRYLING, C. & DE LONGO, J.E. (1980). Transforming growth factors produced by certain human tumor cells: polypeptides that interact with epidermal growth factor receptors. *Proc. Natl Acad. Sci. USA*, **77**, 5258.

VIGNON, F., CAPONY, F., CHAMBON, M., FREISS, G., GARCIA, M. & ROCHEFORT, H. (1986). Autocrine growth stimulation of the MCF-7 breast cancer cells by the estrogen regulated 52 kDa protein. *Endocrinology*, **118**, 1597.

WANG, D.Y., HAMED, H., MOCKRIDGE, C.I. & FENTIMAN, I.S. (1989). Radioimmunoassayable insulin-like growth factor-I in human breast cyst fluid. *Eur. J. Cancer Clin. Oncol.*, **25**, 867.

WESTLEY, B. & ROCHEFORT, H. (1979). Estradiol induced proteins in the MCF-7 human breast cancer cell line. *Biochem. Biophys. Res. Commun.*, **90**, 410.